# Crafting study tasks among medical students: the role of authentic medical teachers

**DOI:** 10.1186/s12909-020-02337-5

**Published:** 2020-11-16

**Authors:** Tuan Trong Luu, Thao Thanh Vo

**Affiliations:** 1 Swinburne Business School, Swinburne University of Technology, John Street, Hawthorn, Victoria 3122 Australia; 2 University of Economics Ho Chi Minh City, Ho Chi Minh City, Vietnam

**Keywords:** Medical teacher, Authentic leadership, Task crafting, Promotion focus, Vietnam

## Abstract

**Background:**

Though leadership has been highlighted as a salient skill for medical teachers in the medical education literature, the role of authentic leadership style among medical teachers in activating medical students’ learning behaviors has not been explored. Our study seeks to examine the effects of medical teachers’ authentic leadership on study task crafting behaviors among medical students.

**Methods:**

Our study adopted a mixed-methods research design comprising observations of 100 surgical operations and 100 ward conferences led by medical teachers, and surveys on authentic leadership, study task crafting, and promotion focus. Structural equation modelling was utilized in the data analysis.

**Results:**

Medical teachers’ authentic leadership demonstrated positive effects on the two study task crafting behaviors (seeking resources (B = 0.36, *p* < 0.001) and seeking challenges (B = 0.21, *p* < 0.05)) but not on reducing study task demands (B = 0.11, *p* > 0.10). Promotion focus was found to strengthen such positive effects of authentic leadership on seeking resources (B = 0.23, *p* < 0.05) and seeking challenges (B = 0.18, *p* < 0.05). Illustrative excerpts of intraoperative and conference conversations are presented.

**Conclusions:**

Our study provided empirical evidence that medical students guided by authentic teachers expressed increased levels of study task crafting, which were further increased if medical students were promotion-focused.

## Background

Medical teachers play various roles in their career. They are medical experts, instructors, mentors, and leaders in the eyes of their students [1]. Nonetheless, while the extant literature has underscored the leadership role of medical doctors in their daily professional activities [2], it has devoted inadequate scholarly attention to the leadership role of medical teachers. Medical students’ engagement in daily study tasks in the clinical environment can be influenced by their medical teachers’ leadership style. When it comes to the leadership style of medical professionals, research has tended to center around transformational leadership [3]. Notwithstanding exerting some effects on follower behaviors [3], transformational leaders tend to view followers secondary to organizational goals [4] while authentic leaders place followers center-stage [5]. Authentic leadership refers to leading people through the modelling of self-awareness, relationship transparency, internalized moral perspective, and balanced processing of information [6] (p. 94). Therefore, adopting authentic leadership, medical teachers are able to both adopt learner-centered approach and help their students acknowledge their limitations, seek inputs, and act morally in their interactions with and service toward patients. Our study seeks to bridge the gap in the medical education literature in terms of the role of authentic leadership among medical teachers.

In their daily study in the hospital, medical students have to engage in clinical study tasks such as collecting and recording patients’ medical history, monitoring and reporting disease progress, providing advice for patients, participating in surgical operations, presenting special cases among their study group, and participating in ward conferences at department and hospital levels. Like members in any organizations, medical students can craft their study tasks to experience more meaning in them. From Tim et al.’s [7] view of task crafting, study task crafting can be defined as changes that students proactively make to the study task resources (e.g., seeking knowledge, skills, and feedback), challenges (e.g., partaking in new study projects), and demands (e.g., reducing role conflicts). Being transformed by authentic leaders into authentic members, medical students realize their limitations, seek resources to improve upon them, seek challenges to further improve their clinical skills, and find ways to alleviate task demands in their interactions with patients. Expressed differently, adopting authentic leadership style, medical teachers can influence their medical students to proactively craft their study tasks to find their medical study and practices meaningful.

Through delving into the nexus between medical teachers’ authentic leadership style and medical students’ study task crafting behavior, our research can contribute to the relevant literature in at least three ways. First, our inquiry can bridge the gap on the scant research on leadership style among medical teachers in general and their authentic leadership style in particular albeit this leadership style is relevant to learner-centered approach. Second, our study advances the learning behavior literature by delving into medical students’ study task crafting behavior as well as the role of medical teachers’ authentic leadership style in activating their students’ task crafting. Third, medical students with different levels of promotion focus may react differently to their teachers’ authentic leadership. Promotion focus is defined as individual inclination to frame their goals around gain and growth [8]. Promotion-focused students display a learning orientation and openness to new experiences [8]. Our research further advances the task crafting research by unravelling the moderating role of promotion focus on the impact of medical teachers’ authentic leadership on study task crafting among medical students.

## Conceptual framework

### Authentic leadership

Authentic leadership is a bottom-up leader behavior pattern that reflects the leader’s self-awareness, internalized moral perspective, balanced information processing, and transparency in their working relation with their followers [6]. Authentic leaders are aware of their values, motives, and limitations, welcome and solicit inputs from others, and share information [9]. They are genuine in acknowledging their followers’ strengths and helpful in stretching their thinking [10]. Authentic leaders represent a social support resource for followers to build their own resources, including values (e.g., self-awareness, self-transcendence) and competencies [11].

### Authentic leadership and study task crafting

Task crafting refers to alterations that individuals make to their task to enhance its meaningfulness for themselves [12]. When crafting their task, individuals seek resources (knowledge, skills, support, and feedback) and challenges, as well as reduce task demands (cognitively or emotionally intense tasks) [13].

Authentic leaders shape authenticity among their subordinates through modelling and transferring a sense of responsibility toward others [14]. Hence, in light of social learning theory [15], medical students are inclined to emulate their medical teachers’ authentic behaviors in their study of medical practices. Working with authentic medical teachers, medical students are inclined to develop a profound sense of self-awareness, thereby acknowledging their limitations in terms of medical knowledge and skills as well as empathetic value in their daily interactions with patients. They tend to be more open to the feedback from teachers, peers, and patients and acquire knowledge from them to improve upon their limitations. Furthermore, authentic leaders tend to stretch the thinking among their followers [10]. Under the supervision of authentic medical teachers, medical students have the propensity to seek opportunities to engage in challenging study tasks to stretch their competence.

Moreover, through relational transparency [6], authentic medical teachers may encourage their students to speak their mind in terms of difficulties or cognitive and/or emotional intensity in their study tasks. With internalized moral perspective [6], authentic teachers have the inclination to help their students reduce their learning task demands in an ethical manner such as seeking support (from peers or nurses) to develop smooth interactions with patients rather than avoiding communication with them. In light of this reasoning, we can expect authentic leadership among medical teachers to shape study task crafting among medical students.

### Promotion focus as a moderator

Individual attributes may function as a boundary condition for individual reactions to leader behaviors [16]. Therefore, students with different levels of promotion focus may perceive authenticity in their medical teacher’s leadership in a different manner. Promotion focus refers to an individual inclination to frame their goals around gain and growth [8]. In comparison with students low in promotion focus, promotion-focused students may more strongly perceive that values from authentic leader behavior can help them gain more resources (knowledge, skills, and feedback) as well as grow morally into authentic physicians. Promotion-focused students are therefore more inclined to emulate authentic behavior than students with low promotion focus, thereby more likely to engage in seeking resources and challenges as well as finding ways to alleviate learning task demands. This line of discussion leads us to anticipate the moderating role of promotion focus in strengthening the impact of medical teachers’ authentic leadership on their students’ study task crafting behaviors.

## Methods

### Data collection

We studied 100 ward conference meetings and 100 surgical operations selected randomly from the departments of general surgery (28 operations), thoracic surgery (11 operations), neurosurgery (8 operations), orthopedic surgery (7 operations), urology (9 operations), oncology (23 operations), and obstetrics and gynecology (14 operations) at a large interdisciplinary hospital in Ho Chi Minh City, Vietnam. This sample size surpassed the benchmark of 97, the necessary sample size to detect medium-level effect sizes at a power level of 0.09 [17]. Participant observations were conducted on ward conference meetings. Intraoperative time was videorecorded and these videos were removed in 60 days as per protocol. The transcription of the videos was conducted by two researchers. Ethics approval was obtained from the Public Health Research Ethics Committee. Instances of leadership behaviors were recorded. At post-conference or postoperatively medical students were asked to rate authentic leadership, task crafting behaviors, and promotion focus.

### Instruments

Students were invited to rate the frequency of authentic behaviors that their medical teacher displayed through Walumbwa et al.’s [6] 16-item Authentic Leadership Questionnaire (1 = never, 5 = almost always) including four dimensions: self-awareness, relational transparency, internalized moral perspective, and balanced information processing (Cronbach’s α = 0.82). Petrou et al.’s [13] scale was adapted to measure four study task crafting behaviors comprising seeking resources (clinical knowledge and skills, support, and feedback) (Cronbach’s α = 0.86), seeking challenges (Cronbach’s α = 0.81), and reducing study task demands (Cronbach’s α = 0.84). Promotion focus was measured through nine items from Neubert et al. [18] (Cronbach’s α = 0.87).

### Coding procedure

De-identified transcripts rather than the primary videos were utilized for analyses to mitigate bias that might result from subject recognition. Two medical researchers who had experience in qualitative research were invited to participate in the transcription process. The first researcher conducted the transcription; the second researcher checked the fidelity of the transcripts and discussed with the first to resolve any mismatches between the videos and the transcripts; and they both then de-identified the transcripts. An author and a management scholar conducted independent reviews of de-identified transcripts of the videos and ratings of medical teachers’ leadership behaviors using authentic leadership scale items.

The score for authentic leadership of each medical teacher was the mean of the subscale scores (self-awareness, relational transparency, internalized moral perspective, and balanced information processing) [6], which were computed by averaging the scores of subscale item scores. Authentic leadership score for each medical teacher was the mean of the scores from the two raters.

## Results

### Survey results

The percentages of male and female medical teachers were 72.2 and 27.8% respectively. Their average age was 47.3 years and their average experience was 23.3 years. Their educational levels included bachelor of medicine (5.6%), masters’ degree of medicine (55.6%), level-1 specialist degree (11.1%), level-2 specialist degree (22.2%), and PhD degree (5.6%). In the medical student sample, the percentages of male and female students were 63.9 and 36.1% respectively. Their average age was 25.9 years and their average experience was 3.9 years. Their educational levels included undergraduate (52.8%) and postgraduate (47.2%).

T-test results indicated no significant differences were found in authentic leadership scores from surveys and observations (t = 1.14; *p* = 0.24). There were no significant differences between observational and survey data for the three task crafting behaviors (Seeking resources: t = 0.84, *p* = 0.27; Seeking challenges: t = 0.71, *p* = 0.29; Reducing task demands: t = 0.62, *p* = 0.31). Nevertheless, a marginally significant difference existed for promotion focus (t = 1.02, *p* = 0.08 < 0.10).

There were the positive associations between authentic leadership and study task crafting behaviors including seeking resources (B = 0.36, *p* < 0.001) and seeking challenges (B = 0.21, *p* < 0.05). Nonetheless, no significant correlation was found between authentic leadership and reducing task demands (B = 0.11, *p* > 0.10). Furthermore, the interaction terms of authentic leadership and promotion focus for seeking resources and seeking challenges were positive and significant respectively (B = 0.23, *p* < 0.05; B = 0.18, *p* < 0.05), which indicates the moderating role of promotion focus in strengthening the effects of authentic leadership among medical teachers on their students’ three study task crafting behaviors. The interaction term of authentic leadership and promotion focus for reducing study task demands was non-significant (B = 0.08, *p* > 0.10).

### Observation results

As an illustration, medical teacher T8 with the highest authentic leadership score expressed authentic attributes since walking into the operating room. This medical teacher highlighted the mission of saving patients (internalized moral perspective) as well as demonstrated his self-awareness, relational transparency, and balanced processing to his students who would assist his operation:

*Medical teacher (Surgeon): Have you had a chance to assist an operation on this kind of tumor?*

*Medical student 1: No, this is my first case.*

*Medical teacher: This is a rare case and this is my sixth chance to handle it [self-awareness]. During the operation, if you have any different idea from my surgical decision, feel free to put it forward [relational transparency].*

*Medical student 2 (Assistant surgeon): Do you still think it is a carcinoma? And can we save the patient after this operation?*

*Medical teacher: It appears to be a carcinoma* via *MRI image analysis. I agree with you that it is too early to conclude it before its biopsy [balanced processing of information]. This is a tough case but I believe that with all our efforts, we can save and have to save the patient from this disease [internalized moral perspective]. Anesthesia expert, have you prepared for bleeding that may occur?*

*Anesthetist: Definitely. I have collected enough blood units for such a situation.*

The medical teacher further solicited some surgical tactics from the students (balanced information processing), thereby activating their task crafting actions:

*Medical teacher (Surgeon): As the MRI image shows, I think we should approach the tumor from the right? What do you guys think? [balanced processing of information].*

*Medical student 2 (Assistant surgeon): From the left, I reckon, to avoid touching the main vessel. Do you think it is a better cut? [seeking feedback].*

*Medical teacher: Excellent.*

*Medical student 2: May I ask a further question? In this case, in order to minimize the dropping of tumor cells after incision, should we adopt procedure A or B? [seeking structural resources (knowledge)].*

*Medical teacher: Procedure A is the best.*

*Medical student 2: Thank you.*

*Medical teacher: [turning to medical student 1] It seems you want to say something.*

*Medical student 1: I would like to have chance to assist you to clamp the vessels while you are removing the tumor. Today I am a circulating tech, but can I swap my role with S (scrub tech) to have a chance to engage in this task? [seeking challenges] S agreed with me [gaining social resources]. I know this is a challenging task for me a medical student, but I believe I can do.*

*Medical student 2: I believe you can do that. I have worked with you in several challenging cases. [providing social resources].*

*Medical teacher: I am happy to give you this chance. I will assist you too. [providing social resources].*

The above excerpt indicates that the most authentic medical teacher created the climate in which medical students were inclined to seek structural (knowledge) and social resources (feedback and support) as well as challenges. Furthermore, authentic medical teachers helped students surmount their cognitive or emotional task demands as in the case of medical teacher T17 below:

*Medical teacher: This is a tough case for your group project. However, I encourage you to handle it. Lots to learn. [relational transparency].*

*Medical student A: We have read about this case, but there are lots of signs on this patient we are unable to explain, for instance, why does her ECG show negative T wave?*

*Medical teacher: I have prepared this article for you. You can find an explanation in it. [helping reduce cognitive task demand].*

*Medical student B: One more thing. This patient is too hard to communicate with. We are struggling with exploring all symptoms from her.*

*Medical teacher: Does she not like you all? [smiling] We should empathize with patients. I remember Miss C (medical student) can talk with her a bit. Let C kickstart the conversation with her. I have also asked Miss N (nurse) to help you when you approach this patient. [helping reduce emotional task demand].*

On the contrary, medical teacher T42, the teacher with the lowest authentic leadership score, demonstrated his lack of moral value and care for patients:

*Medical teacher: I don’t know why I was asked to operate on this scheduled patient. This is not mine. I will just remove the tumor and close the abdomen, that’s it, though I may find other problems in it.*

The surgeon further created a constrained work climate, which did not encourage students to develop study task crafting behaviors:

*Medical teacher: Remember that you are here to assist my operation. Just follow my instructions and please don’t disturb me with questions about the disease. You can learn about the disease through your books and journal articles.*

## Discussion

The findings unpacked the positive relationships between authentic leadership among medical teachers and medical students’ engagement in study task crafting behaviors including seeking structural and social resources and seeking challenges. However, the empirical support was not found for the link between authentic leadership and task demand reducing behavior. This is probably because medical students in the Vietnamese context, who had been accustomed to intensely cognitive tasks in their study from primary to tertiary education levels [19], might have high levels of tolerance of high cognitive intensity in their medical study and might not seek ways to alleviate such study task demands. Moreover, growing up in a collectivistic culture, Vietnamese medical students were inclined to develop empathetic concerns for patients with their pains rather than finding ways to avoid emotionally intense interactions with demanding patients. The results also unveiled the role of promotion focus in strengthening the effects of authentic leadership on resource seeking and challenge seeking behaviors among medical students.

Through these findings, our study can cover some gaps in the medical educational leadership literature. First, researchers have underscored the magnitude of leadership development among medical professionals as well as teachers [1]. Yet, compared with research on leadership among educators or clinicians at managerial positions in healthcare educational institutions, empirical research on leadership among medical teachers has been in its infancy [1].

Moreover, research has tended to view clinical leadership via the lens of transformational leadership framework. Horwitz et al.’s [20] survey findings provided support for the role of surgeons’ transformational leadership in fostering team members’ perception of satisfaction and extra efforts. Through an observational study, Hu et al. [3] reported the effects that transformational surgeons exerted on their team members’ knowledge sharing and voice behaviors.

While transformational leaders place organizational goals first and members second [4], authentic leaders tend to put members center-stage [5]. Therefore, authentic leadership seems relevant to the role of medical teachers in nurturing expertise and moral values among medical students. Authentic medical teachers are likely to create authentic medical experts who can acknowledge their limitations and improve upon them, share their knowledge with others, and behave morally especially toward patients. Our research, to our best knowledge, is the first to investigate the role of authentic leadership among medical teachers, thereby advancing the leadership research stream in the medical education literature.

Second, our study also extends the medical education literature by delving into study task crafting behaviors among medical students. Medical students learn clinical skills mainly through study tasks in their daily interactions with patients. Nonetheless, medical education research still has remained quiet about study task crafting behaviors. Our research pioneers to advance the concept of task crafting from the generic management literature to the medical education sphere. Furthermore, our inquiry provided empirical endorsement for the nexus between medical teachers’ authentic behavior and medical students’ study task crafting.

Third, medical student behaviors tend to be the function of contextual factors and individual factors. Nevertheless, the medical education literature has tended to consider the impact of either contextual factors (leadership) or individual factors (student attributes) on student behaviors rather than investigating the simultaneous effects of these factors. Our research initiates the exploration into the interactive effects of authentic leadership among medical teachers and promotion focus inside medical students. Promotion-focused medical students, who frame their study goals around gain and growth [8], are inclined to respond more strongly to authentic medical teachers and develop authenticity in their learning behavior, thereby proactively seeking resources for crafting their study tasks.

Finally, clinical leadership research has largely been conducted via surveys with very few exceptions of observational studies [3]. Our research on medical teacher leadership has surmounted this methodological limitation of prior research by adopting a method triangulation approach including surveys and observations of intraoperative and intra-conference interactions among medical teachers and medical students.

## Limitations and future research

First, our research collected authentic leadership scores for medical teachers through surveys and observations based on Walumbwa et al.’s [6] authentic leadership scale. By virtue of our aim to observe medical teachers behaving as naturally as they can in the operating rooms or in the ward conferences, we analyzed verbatim transcripts of the communication and interactions between medical teachers and their students and compared the observational findings with the survey responses from the students. Since our research has no precedent for observational use of authentic leadership scale, further research should be conducted in this stream to further verify the observational application of this instrument.

Second, despite being conducted at a single hospital, numerous observations helped produce significant variability in medical teachers’ leadership behaviors. Third, albeit leadership style is a relatively stable variable associated with personality [21], anomalous situations in our observed cases might lead medical teachers to atypical reactions that did not represent their baseline leadership styles. Further observational studies are thus required to assess the role of the situational factors in shaping leadership style among medical teachers.

## Conclusions

Our study can serve as a framework for the assessment of teacher leadership in medical education by providing mixed-methods evidence that medical students working under authentic leadership of medical teachers can develop study task crafting behaviors. Our research highlights the salience of leadership in medical teachers in building generations of medical experts who can “lead” their colleagues in their daily clinical activities.

## Data Availability

The data that support the findings of this study are available from University of Economics Ho Chi Minh City but restrictions apply to the availability of these data, which were used under license for the current study, and so are not publicly available. Data are however available from the authors upon reasonable request and with permission of University of Economics Ho Chi Minh City.

## Abbreviation

B: Beta
